# Ectopic osteogenesis and angiogenesis regulated by porous architecture of hydroxyapatite scaffolds with similar interconnecting structure in vivo

**DOI:** 10.1093/rb/rbw031

**Published:** 2016-09-20

**Authors:** Jinyu Li, Wei Zhi, Taotao Xu, Feng Shi, Ke Duan, Jianxin Wang, Yandong Mu, Jie Weng

**Affiliations:** ^1^Key Laboratory of Advanced Technologies of Materials (MOE), School of Materials Science and Engineering, Southwest Jiaotong University, Chengdu 610031, China; ^2^Dental Department, Sichuan Province People’s Hospital, Chengdu 610072, People’s Republic of China

**Keywords:** : hydroxyapatite scaffolds, similar interconnecting structure, osteogenesis, angiogenesis

## Abstract

The macro-pore sizes of porous scaffold play a key role for regulating ectopic osteogenesis and angiogenesis but many researches ignored the influence of interconnection between macro-pores with different sizes. In order to accurately reveal the relationship between ectopic osteogenesis and macro-pore sizes in dorsal muscle and abdominal cavities of dogs, hydroxyapatite (HA) scaffolds with three different macro-pore sizes of 500–650, 750–900 and 1100–1250 µm were prepared via sugar spheres-leaching process, which also had similar interconnecting structure determined by keeping the d/s ratio of interconnecting window diameter to macro-pore size constant. The permeability test showed that the seepage flow of fluid through the porous scaffolds increased with the increase of macro-pore sizes. The cell growth in three scaffolds was not affected by the macro-pore sizes. The in vivo ectopic implantation results indicated that the macro-pore sizes of HA scaffolds with the similar interconnecting structure have impact not only the speed of osteogenesis and angiogenesis but also the space distribution of newly formed bone. The scaffold with macro-pore sizes of 750–900 µm exhibited much faster angiogenesis and osteogenesis, and much more uniformly distribution of new bone than those with other macro-pore sizes. This work illustrates the importance of a suitable macro-pore sizes in HA scaffolds with the similar interconnecting structure which provides the environment for ectopic osteogenesis and angiogenesis.

## Introduction

In bone tissue engineering, porous three-dimensional scaffolds have been used extensively as the template for cell interactions and the formation of extracellular bone matrix to provide structural support to newly formed tissue. The properties of scaffolds are closely related to initial cell–scaffold interactions, angiogenesis and subsequently induction of tissue formation [1–3]. These properties are primarily dependent on the nature of the material and the manufacture process. The nature of various material has been extensive studied, such as bioceramics [4, 5], metals [6, 7], bioglass [8, 9], polymers [10, 11] and composite materials [12, 13]. Additionally, architectural characteristics (e.g. geometry, size, volume fraction and inerconnectivity of pores) are also critical to scaffolds, and has been used to improve the performance in tissue engineering [14].

The architecture characteristics of scaffold are important for regulating cell reaction and enhancing the efficiency of tissue ingrowth [15]. The porous structure not only serves as template for cell adhesion and the new bone formation [14, 16] but also acts as a pathway for the transport of nutrients and removal of waste. Therefore, an appropriate porous structure design in scaffold is necessary to tissue regeneration. Earlier studies reported that pore geometry in scaffolds strongly influenced cell behaviors and tissue growth [17]. The amount of tissue deposited was found proportional to the curvature of macro-pores [18]. Foam-shaped scaffolds with spherical pores showed a higher compressive strength than those having strip-shaped pores [17]. Moreover, a higher porosity in scaffold did not affect cell attachment but improved cell proliferation and transport of oxygen and nutrients [19]. Numerous studies revealed that a higher porosity could enhance tissue ingrowth and new bone formation after implantation in the rabbit cranium [20]. In addition, the inerconnectivity of macro-pores was observed to determine whether new tissues sequentially penetrated from the surface into deeper pores through the interconnected pores [21]. Other studies found that, when macro-pores were not interconnected, the angiogenesis was restrained even though the volume porosity was high [14, 22]

In recognition of the importance of architectural characteristics, studies have attempted to tailor the macro-pore sizes as key parameter to control cell response [23, 24], angiogenesis [25] and tissue ingrowth [26]. Previously studies showed that compared with one having lager pores (200–400 μm), scaffolds with smaller macro-pores (50–100 μm) offered a much larger surface available to the invading tissue elements [27], and macro-pores diameter of > 100 μm was not required for bone ingrowth [28]. Other studies suggested that the macro-pore size should be at least 100 μm in diameter for successful diffusion of essential nutrients and oxygen for cell survival [29]. Macro-pores < 100 μm were penetrated only by fibrous tissues after implantation in the canine femur [30]. Porous HA ceramics with different macro-pore sizes in the range of 106–600 μm were fabricated to evaluate the tissue growth after implantation in rats, and a macro-pore size of 300–400 μm was found most effective for osteogenesis and angiogenesis [31, 32]. Additionally, other works observed cell colonization and new bone formation was improved in the macro-pore size of 565 μm compared with 300 μm [33]. Yet other studies observed larger macro-pores (800 μm) in the tissue engineered constructs yielded more bone than those with smaller ones (400 μm) after ectopic implantation in goats [34]. Thus, there is no consensus about the optimal macro-pore size of scaffolds for vascularization and tissue growth, and many researches just focus on the change of macro-pores sizes and ignored the influence of interconnecting window diameters between macro-pores with different sizes in the process of bone formation. Some works have demonstrated that the interconnecting windows affected not only the sizes and number of the blood vessels formed but also the efficiency of osteogenesis in porous scaffolds [35, 36]. Therefore, to compare the influence of macro-pore size on osteogenesis and angiogenesis is necessary to consider simultaneously the effect of interconnecting window diameter.

Hydroxyapatite (HA) is an excellent biomaterial and porous HA scaffolds have been demonstrated to promote osteogenesis and angiogenesis by inducing tissue growth into their pores [37, 38]. In this study, porous HA scaffolds with different macro-pore sizes in ranges of 500–650, 700–950 and 1100–1250 µm were developed by combining gel-casting, porogens-leaching. The scaffolds with different macro-pore sizes represented similar interconnecting structure which determined by the almost 0.26 ratio of interconnecting window diameter to macro-pore size via heat treatment technique. As a result, all kinds of scaffolds were maintained similar interconnecting structure. The effects of macro-pore sizes on osteogenesis and angiogenesis in porous HA scaffolds were investigated in a canine model.

## Materials and methods

### Materials

HA powder with average mean size of 20 μm was synthesized in our laboratory through the wet-chemical method. N, N-dimethylacetamide (DMAc), lithium chloride (LiCl), chitin, dimethyl benzene, n-hexane, acetone and all other chemicals used in the preparation of porous HA scaffolds were of analytic grade and purchased from Chengdu Chemical Engineering Factory (Sichuan, China). Sucrose sugar and vegetable oil were purchased from a local super market.

### Preparation of sugar spheres

Sugar spheres with different sizes were prepared by an emulsion process. Briefly, 60 g of sucrose were melted at 100°C to give a clear yellowish liquid. The liquid was added into 200 ml of vegetable oil (preheated to 160°C) under mechanical stirring, and the mixture was stirred for 30 min to form a suspension. Then, 200 ml of pre-cooled vegetable oil was poured into the suspension. After discarding the vegetable oil, sugar spheres were collected, rinsed four times with hexane and sieved to select desired sizes. Identical sugar spheres were packed in a cylindrical mold and heat-treated for certain time at 70°C to form template. the different heat-treated time for various templates which were made of sugar spheres different sizes, could be make sure the scaffolds with different macro-pore sizes possessed the same d/s ratio of interconnecting window diameter to macro-pore size, which were summarized in Table 1.

**Table 1:** Structural parameters of HA scaffolds after the sugar spheres templates for heat treated different time at 70 °C

Macro-pore size (μm)	Heating time for sugar spheres (min)	The diameter of interconnecting windows (μm)	The ratio of window diameter to macro-pore size	Porosity (%)
1100–1250	0	174 ± 11.95	0.15 ± 0.03	82.6
1100–1250	8	321 ± 13.94	0.26 ± 0.02	83.5
1100–1250	15	548 ± 21.46	0.48 ± 0.03	84.1
750–900	5	228 ± 12.10	0.26 ± 0.03	83.2
500–650	3	135 ± 9.30	0.25 ± 0.05	82.1

### Preparation of porous HA scaffolds

The fabrication process of porous HA scaffolds have been described elsewhere in detail [39]. Briefly, 0.75 g of chitin was dissolved in 100 ml of a 5% LiCl/DMAc solution till the liquid became transparent. The 25 g of HA powder was followed dispersed in the liquid and stirred for 6 h to form a HA/Chitin slurry. The slurry was extruded into the mold containing the sugar spheres templates. The HA/chitin samples were rapidly gelated by soaking in distilled water, during which sugar spheres also dissolved slowly. The HA/chitin samples were dried at 80°C overnight and cut by a razor blade to cylinders. Finally, samples were sintered at 500°C for 2 h to remove the chitin, and sintered at 1200°C for 2 h to form the porous HA scaffolds. The scaffolds with different macro-pore sizes in the ranges of 500–650, 750–900 and 1100–1250 µm were prepared by controlling the sizes of sugar spheres. All the scaffolds with size ofΦ10 × 15 mm were used for mechanical testing and Φ10 × 8 mm were used for other experiments.

### Characterization of HA scaffold

#### Morphological characterization

Morphologies, macro-pores sizes and interconnectivity of HA scaffolds were examined by scanning electron microscopy (SEM, FEI Quanta 200).

#### Mechanical testing and porosity

Compressive strength was tested with an Instron 5567 universal testing machine equipped with a 30 kN load cell at a displacement rate of 0.5 mm/min using Φ10 × 15 mm cylinders (see ‘Preparation of porous HA scaffolds’ section). Five specimens were tested for each group and the load was continued until the scaffold failed. Average porosities were measured by gravimetric method and the Archimedes method with five samples for each group [14].

### Characteristic of seepage flow in scaffolds analysis

The characteristic of seepage flow (fluid flow through porous media) of HA scaffolds with three different macro-pores sizes were analyzed by testing the speed of ink/water mixture flowed though the scaffolds. Briefly, porous HA scaffolds were placed in the middle of centrifuge tubes and sealed their edges with plastic tape. The centrifuge tubes were filled with water until the water level just submerged the scaffolds. Finally, the ink/water mixture was simultaneously poured into the three centrifuge tubes containing scaffolds with three different macro-pores sizes to observe the permeability of ink through porous scaffolds.

### Cell isolation and 3D culture

SD rat calvarial osteoblast (RCO) were isolated from 10-day-old neonatal Sprague Dawley rats [40]. Brieﬂy, Calvaria were excised aseptically, cleaned of contaminating connective tissues, and dissected into fragments. The harvested fragments were rinsed with PBS, and then treated at 37°C with 0.25% (w/v) of trypsin for 15 min. The supernatant of the ﬁrst digestion was discarded and subsequently digested with 700 U/ml of Collagenase type I (Sigma-Aldrich, St Louis, MO, USA). The calvarial fragments were treated five more times with Collagenase type I (20 min at 37°C), and the subsequent supernatants were collected. The supernatant was collected and centrifuged at 1500 rpm for 5 min. The cell pellet was then resuspended in α-MEM supplemented with 10% (v/v) FCS, and incubated at 37°C under the atmosphere of 5% CO_2_ in a T25 culture ﬂask. The medium was replaced every 2 days.

Prior to cell experiments, a sufficient number of the experimental cells were grown by the passaging method. RCOs were expanded until 80–100% conﬂuent and released from tissue culture ﬂasks using a 0.05% trypsin and 0.02 g/l EDTA-Na_2_ solution. The fifth passage RCOs were digested, collected by centrifugation (1200 rpm, 3 min) and dispersed (1.0 × 10^6^ cells/ml) in medium for the use of seeding.

Discs (Φ10 × 8 mm discs, ‘Preparation of porous HA scaffolds’ section) were used for *in vitro* cell culture. After sterilizing with ethyleneoxide fumigation, osteoblast cells were seeded on each disc (2 × 105 cells/disc) and cultured (37°C, 5% CO_2_, 98% relative humidity) for 1, 3 and 7 days, respectively. At end time point, a 3-(4,5-dimethylth-iazol-2-yl)-2, 5-diphenyltetrazolium bromide (MTT) solution was added to each well, and the plates were incubated for 3–4 h. Dimethyl sulfoxide was then added to each well for 5 min, and 200 μl of the solution of each well was added to a new 96-well plate. The optical density value was measured at 570 nm by using an automated plate reader.

Discs for each material, at the end of the experiment, were processed for SEM (Eindhoven, The Netherlands): RCOs grown on the materials were washed with PBS buffer, ﬁxed in 2.5% glutaraldehyde solution for 30 min and then dehydrated with graded ethanol solutions (25, 50, 75, 95 and 100%, 15 min each concentration). Dehydrated discs were dried by a vacuum dryer before SEM examination.

### Animal implantation experiments

Nine healthy dogs (1-year-old, 17–20 kg) were randomly divided into three groups according to type of scaffold implanted. All surgical procedures involving animals were approved by the Animal Care and Use Committee of Southwest Jiaotong University. The HA scaffolds with different macro-pore sizes were implanted in the animals for 1, 3 and 6 months. After anesthesia by intravenous injection of 3 g of sodium pentobarbital in 100 ml physiological saline, scaffolds of each kind were implanted in the dorsal muscles and abdominal cavities of dogs. The dorsal thoracolumbar region was shaved, washed and disinfected. An incision was made on the skin along the dorsal midline from T8 to L5 to expose the paraspinal muscles. Nine intramuscular pockets were created in the bilateral dorsal muscles along the vertebra by blunt dissection. Scaffolds with macro-pore sizes in the ranges of 500–650, 750–900 and 1100–1250 µm (three samples each group) were randomly inserted in each pocket. The wound was closed in layers after sufficient rinse with normal saline. Then, the dog was turned to a supine position, and the abdominal region was shaved, washed and disinfected. Incisions were made through the skin parallel to the groins to expose the abdominal muscles, and then the peritoneum was opened by blunt separation. Nine scaffolds with macro-pore sizes in the ranges of 500–650, 750–900 and 1100–1250 µm (three sample each group) were randomly placed in the pockets and sutured to the parietal peritoneum, ensuring a sufficient contact with the parietal peritoneum and omentum. The peritoneum and wound were closed layers by layer. The dogs were given intramuscular penicillin injection (803104 IU) by for 3 d to reduce the risk of postoperative infection.

### Histological analysis

All the dogs were euthanized by sodium pentobarbital overdosing at 1, 3 and 6 months after implantation. The scaffolds were harvested, fixed with 4% formaldehyde and demineralized in an ethylenediamine tetraacetic acid solution. After dehydration in an ethanol series, the demineralized samples were embedded in paraffin and cut perpendicular to the longitudinal axis into 5-mm-thick sections in the center of the implant.

The sections were stained with hematoxylin/eosin (HE) and Masson’s trichrome and studied by optical microscopy (Nikon TS-100). Area percentage of total newly formed bone (%bone) and vessel density in each sample was quantitative measured using Image Pro Plus 6.0 to evaluate the effects of macro-pore sizes on osteogenesis and angiogenesis. Five regions were randomly selected from each section for the analysis.

### Statistical analysis

Data were compared by *t*-test, and a *P* values < 0.05 as considered statistically significant. In following sections, data are expressed as mean ± SD.

## Results

### Characterization of HA scaffold

**Figure 1.**
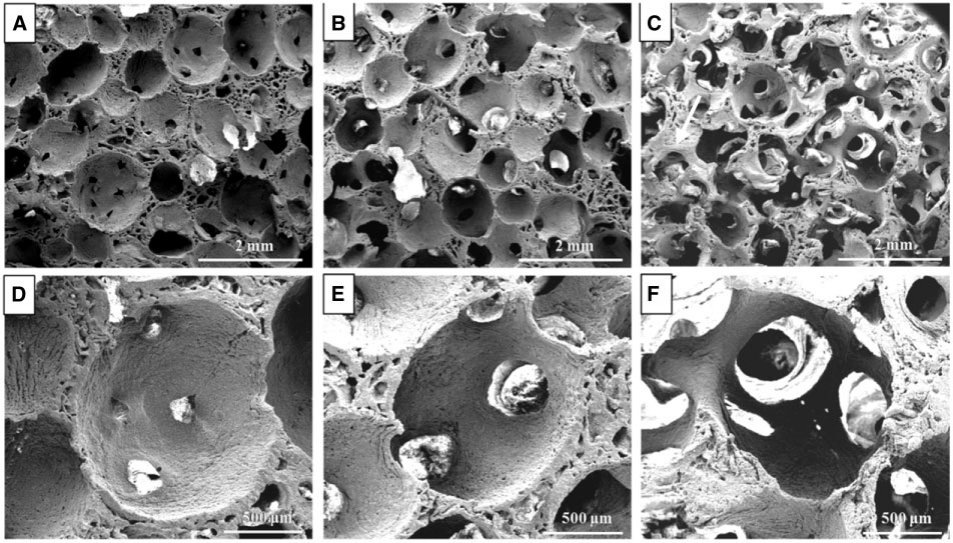
SEM Micrographs of HA scaffolds with macro-pore size in the ranges of 1100–1250 µm after the sugar spheres templates for different heat treated time at 70 °C: **(A)** without heat treatment; **(B)** 8 min; **(C)** 15 min; **(D–F)** are enlarged figures of (A–C), respectively

Numerous uniformly distributed spherical macro-pores were observed in the porous HA scaffolds prepared using sugar spheres template-leaching technology (Fig. 1). The porous scaffolds prepared from the sugar spheres templates without heat treatment showed a certain amount of closed macro-pores (Fig. 1A and D). For these scaffolds prepared from heat-treated sugar spheres templates, there was more than one interconnecting window on the walls of each macro-pore. The result revealed that the HA scaffolds possessed excellent interconnectivity after heat treatment. Besides, the diameters of interconnecting windows increased with the duration of the heat treatment time for templates (Fig. 1A–C). The mean diameters of the interconnecting windows in HA scaffold with macro-pore size in range of 1100–1250 μm increased from 174 ± 11.95 to 548 ± 21.46 μm when heat treatment time changed from 0 to 15 min, but the porosity of the scaffolds almost remain unchanged as shown in Table 1. Therefore, the diameters of interconnecting window were readily controlled by varying the heat treatment time.

**Figure 2.**
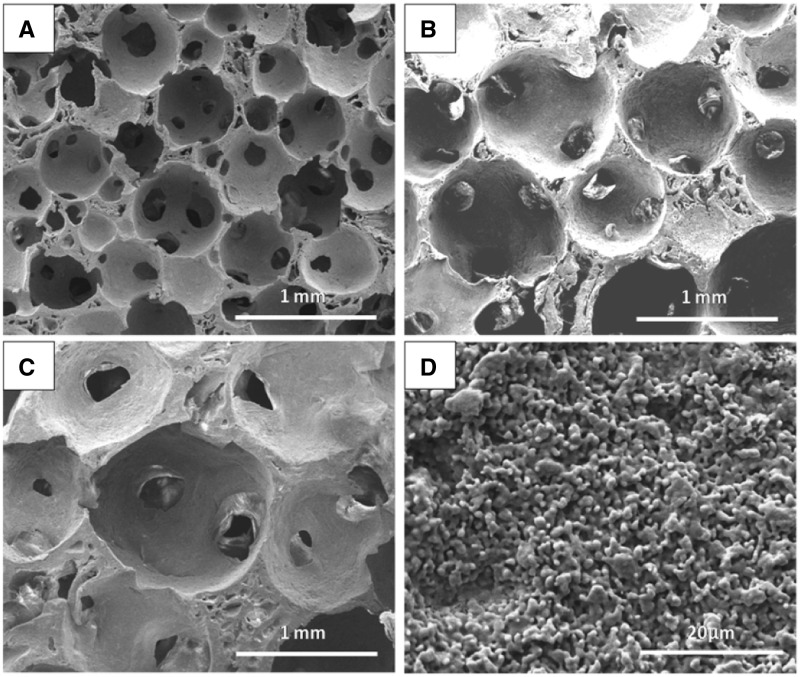
The SEM micrographs of HA scaffolds with the same d/s ratio of interconnecting window diameter to macro-pore size but different pore sizes after the sugar spheres templates heated at 70 °C: **(A)** 500–650 µm for 3 min, **(B)** 750–900 µm for 5 min, **(C)** 1100–1250 µm for 8 min and **(D)** surface topography on the macro-pores wall

**Figure 3.**
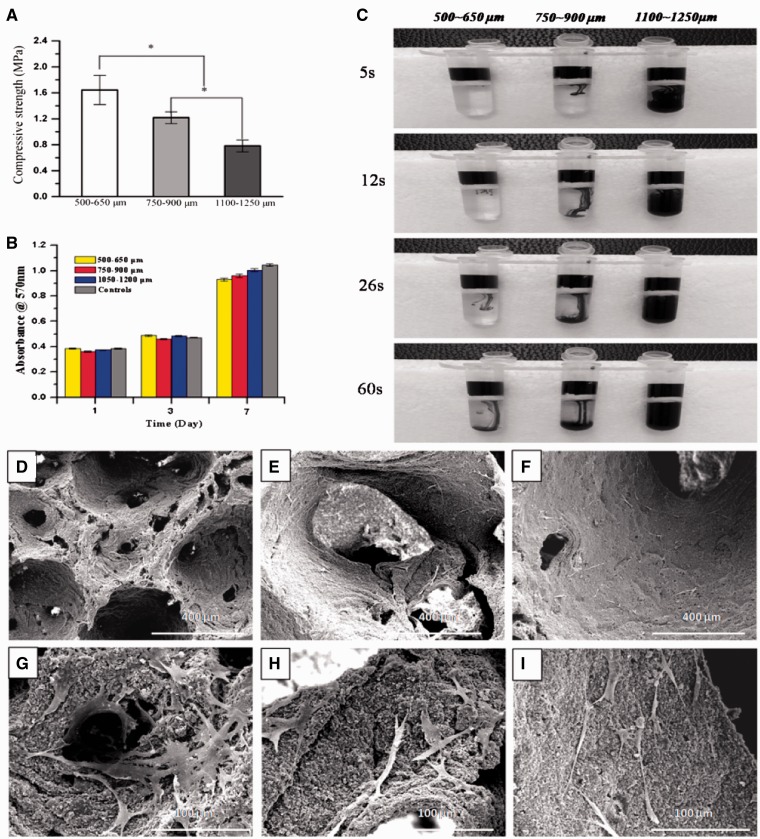
(A) Compressive strengths of HA scaffolds with different macro-pore sizes (mean ± SD, *n* = 5); **(B)** the cell proliferation of scaffolds with three kinds of macro-pore sizes after 7 days in culture; **(C)** the digital camera photographs showing the penetration process of ink through porous HA scaffolds with three macro-pore sizes; cell morphologies on the macro-pore walls of scaffolds with three macro-pore sizes after 7 days in culture: **(D)** 500–650 µm, **(E)** 750–900 µm **(F)** 1100–1250 µm, **(G–I)** are enlarged figures of (D–F), respectively

To avoid the limit of interconnecting windows in different macro-pore sizes and accurately comparing the effect of macro-pore sizes on the bone formation, the fabricated porous HA scaffolds with macro-pore ranges in 500–650, 700–950 and 1100–1250 µm represented similar interconnecting structure (Fig. 2), which determined by the almost 0.26 ratio of interconnecting window diameter to macro-pore size in three macro-pore sizes via adjusting heat treatment time, as showed in Table 1. In addition, much regular micro-pores existed on the wall of macro-pores of all three porous scaffolds (Fig 2D), which are supposed to enhance the attachment, proliferation and differentiation of cells. The compressive strengths decreased with the macro-pores sizes increasing as shown in Figure 3A, although the three scaffolds had the nearly same porosity of 83% (Table 1).

Ink composed of micro-particles of dye was employed to simulate the penetration process of cells-containing body fluid through the pores with three sizes in the HA scaffolds (Fig. 3C). At the first 5 s, ink went completely through the porous scaffold with macro pores of 1100–1250 μm while almost no ink through one of 500–650 μm. There was obviously but small quantity of ink penetrating the HA scaffold of 750–900 μm. The result revealed that ink passed the all type of scaffold successfully with duration of the time, and the speed of ink passing the scaffold increased with the increase of macro-pores sizes (Fig. 3C).

### Characteristic of cell 3D culture

SEM observation showed that the cells were spindle-shaped, uniformly distributed throughout all of the scaffolds and tightly attached to the surface of the porous wall of 3D scaffolds with extended pseudopodia (Fig. 3D–F). Around cells there could be seen some extracellular matrices. Figure 3H–I showed that cells attaching to the macro-pore walls of scaffolds with filopodia. The cell proliferation of each HA scaffolds group was evaluated by MTT at 1, 3 and 7 days, with 2D culture as control group. The results (Fig. 3B) showed that the increasing trend of cell proliferation in all groups was consistent with which of control group and there were no significant differences in cell growth at each culture period.

### Histological characterization of bone ingrowth

No harvested grafts showed inflammation, tissue necrosis, or foreign body reaction, confirming their biological safety in these animals. All the scaffolds retrieved held the physical integrity and were in direct contact with the host tissue.

**Figure 4.**
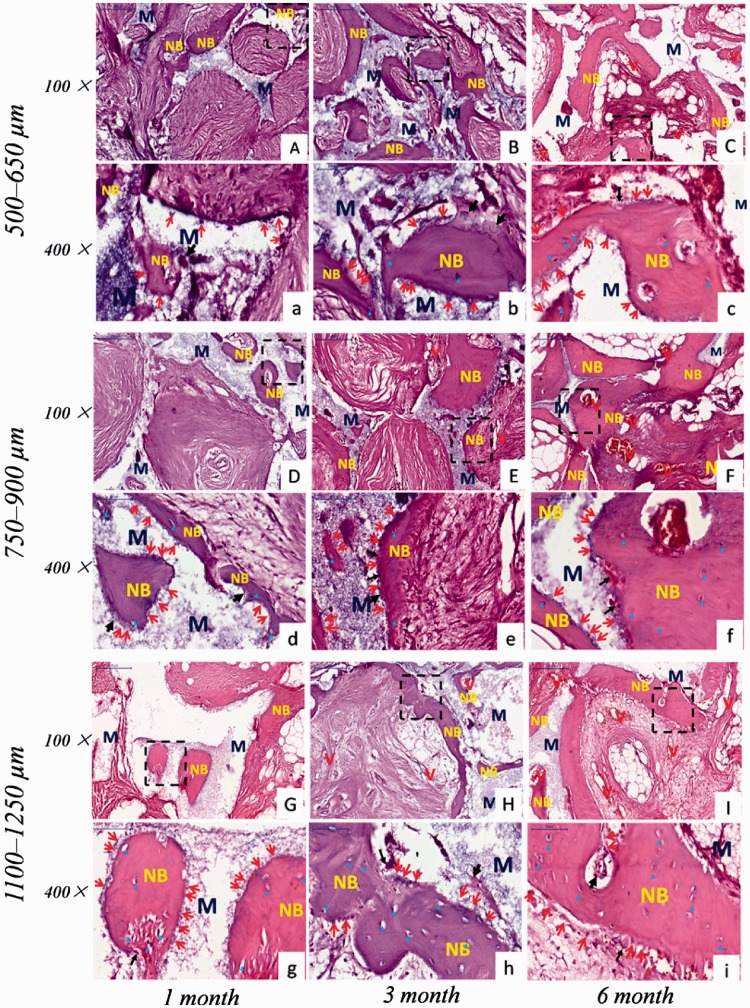
The received HA scaffolds with three pore size ranges implanted in dorsal muscle stained with HE: 500–650 µm **(A–C, a–c)**; 750–900 µm **(D–F, d–f)**; 1100–1250 µm **(G–I, g–i)**. (a–i) are enlarged figures of (A–I), respectively. NB, new bone; M, HA scaffolds; V, new blood vessel; red arrow showed osteoblast; black arrow showed osteoclast; blue delta showed bone lacuna

**Figure 5.**
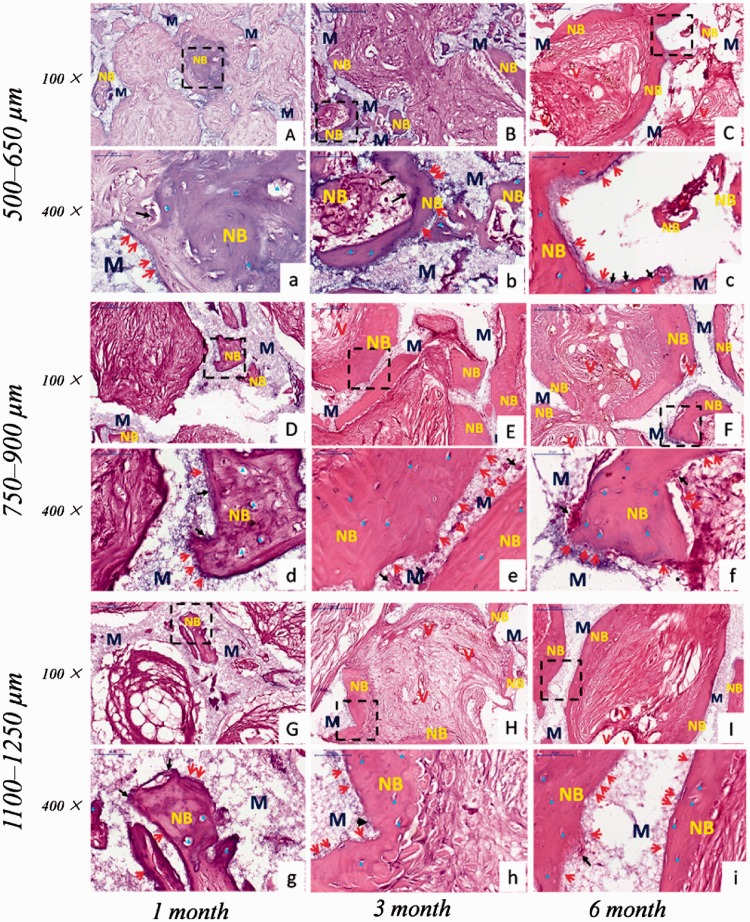
The received HA scaffolds with three pore size ranges implanted in abdominal cavities stained with HE: 500–650 µm **(A–C, a–c)**; 750–900 µm **(D–F, d–f)**; 1100–1250 µm **(G–I, g–i)**. (a–i) are enlarged figures of (A-I), respectively. NB: new bone; M: HA scaffolds; V: new blood vessel; red arrow showed osteoblast; black arrow showed osteoclast; blue delta showed bone lacuna

**Figure 6.**
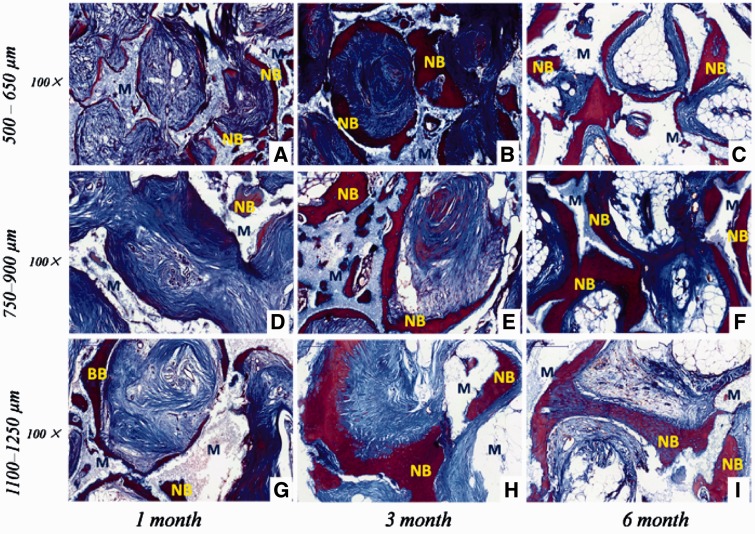
The received HA scaffolds with three macro-pore sizes implanted in dorsal muscle stained with masson trichrome: 500–650 µm **(A–C)**; 750–900 µm **(D–F)**; 1100–1250 µm **(G–I)**. NB, new bone; M, HA scaffolds

**Figure 7.**
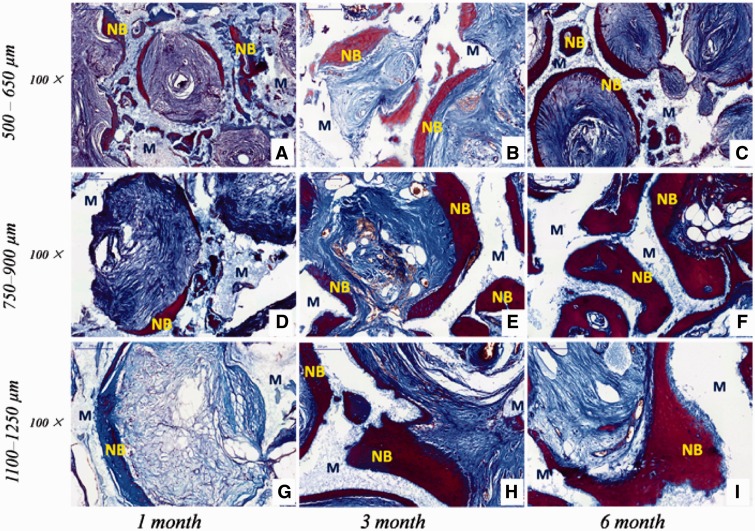
The received HA scaffolds with three macro-pore sizes implanted in abdominal cavities stained with masson trichrome: 500–650 µm **(A-C)**; 750–900 µm **(D–F)**; 1100–1250 µm **(G–I)**. NB, new bone; M, HA scaffolds

Histological examination revealed that new bone and vascularization were observed after implantation regardless of macro-pores sizes and implantation sites. HE staining (Figs 4 and 5) showed that 1 month after implantation whether in the abdominal cavity or dorsal muscle, connective tissues and neovessels grew into the scaffolds and a small amount of new bone tissue was sporadically present (Figs 4A, D, G and 5A, D, G). The blood vessels and new bone tissues increased with the duration of implantation in all scaffolds. After 3 and 6 months implantation, bony callus and new bone tissues clearly appeared in macro-pores and contacted the surface of macro-pores wall both in dorsal muscles and abdominal cavities (Figs 4 and 5). Many osteoblasts and osteoclasts were observed and bone lacuna was presented with formation of new bone tissue (Figs 4a–i and 6a–i). These result demonstrated that biological reconstruction of scaffold were occurring actively and new bone was formed. Masson trichrome staining showed that more lamellar bone was present in scaffolds implanted in the dorsal muscle and in the abdominal cavity after 3 and 6 months implantation (Figs 6 and 7), suggested that calcium deposition was significantly better with the duration of implantation. The difference of formation of new bone and blood vessels in three HA scaffolds was introduced in detail in the following sections.

**Figure 8.**
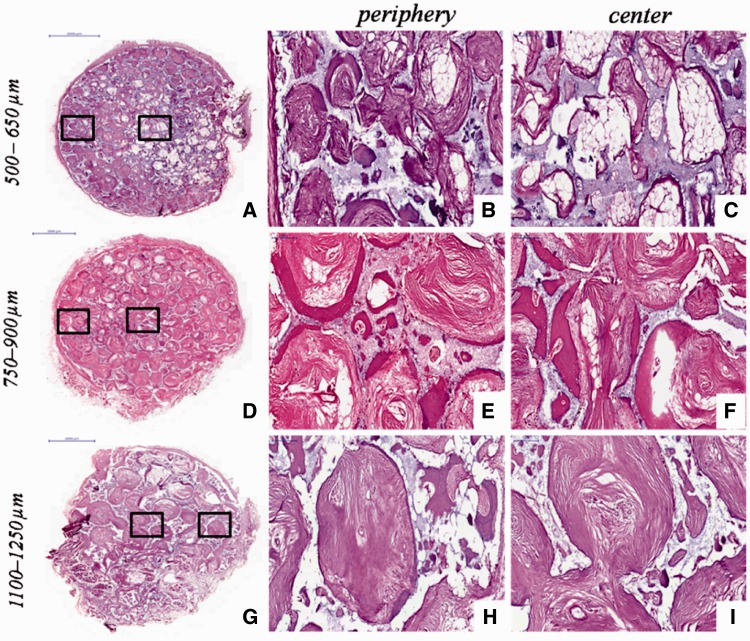
The received HA scaffolds with three macro-pore sizes implanted in dorsal muscle after 3 months stained with HE: 500–650 µm **(a)**; 750–900 µm **(d)**; 1100–1250 µm **(g)**. **(b,c), (e,f) and (h,i)** are enlarged figures of (a), (d) and (g), respectively

In addition, the different new bone distributions in HA scaffolds with three macro-pore sizes implanted in dorsal muscle after 3 months were exhibited by HE staining (Fig. 8). The new bone major distributed in peripheral region of scaffold with macro-pores sizes in 500–650 µm (Fig. 8b), and the center region of scaffold with much blank indicated that the new bone tissue did not growth in (Fig. 8c). The scaffold with macro-pores sizes in 1100–1250 µm exhibited new bone tissue growth in peripheral and center of scaffold (Fig. 8g), but new bone formed was obviously lesser than scaffold with macro-pores sizes in 700–950 µm (Fig. 8h). On the contrary, the tissue obviously occupied all the space in scaffold with macro-pores sizes in 700–950 µm, and the new bone uniformly formed throughout the whole scaffold (Fig. 8d–f).

**Figure 9.**
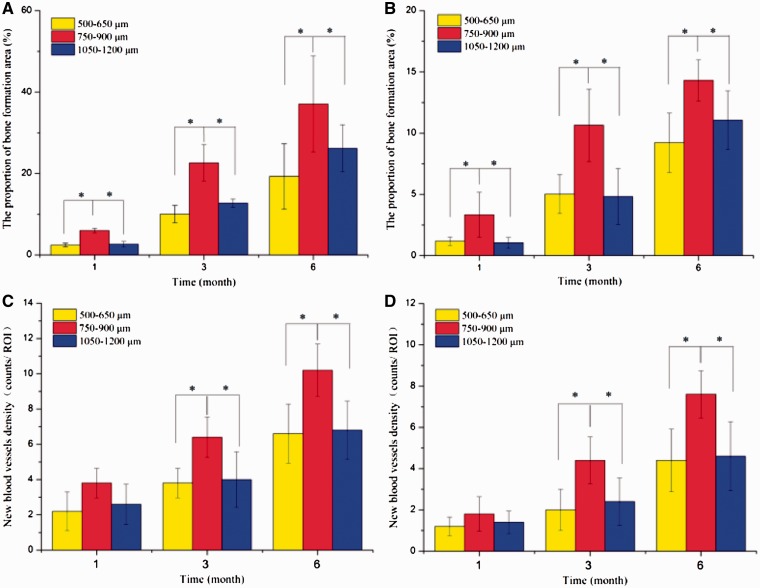
Bone formation and angiogenesis in the HA scaffolds with three kinds of macro-pore sizes, implanted in dorsal muscle, measured at 1, 3 and 6 months. Bone formation: **(A)** implanted in dorsal muscle; **(B)** implanted in abdominal cavities. Angiogenesis: **(C)** implanted in dorsal muscle and **(D)** implanted in abdominal cavities. The error bars represent SDs. An asterisk (*) denotes statistical difference (*t*-test, *P* < 0.05)

Histomorphometric measurement (Fig. 9) showed that, regardless of the site of implantation, the area of new bone infiltration into HA scaffolds increased significantly with time after implantation. After 6 months implantation, the areas of new bone formation (%) were almost 19.30, 37.08 and 26.20% in the dorsal muscle, and were 9.2, 14.30 and 11.06% in the abdominal cavity, respectively with macro-pores sizes in range of 500–650, 700–950 and 1100–1250 µm. The results indicated that the HA scaffolds with macro-pores sizes in range of 750–900 µm implanted in both sites displayed significantly higher total new bone formation than other two kinds of scaffolds (all *P* < 0.05). However, there was no significant difference about new bone formation in 500–650 and 1100–1250 µm. The results also suggested that, new bone tissue formed faster in the dorsal muscles than in the abdominal cavity. Additionally, histomorphometric analysis revealed that the number of blood vessels followed a similar pattern with new bone tissue formation in scaffolds with different macro-pores sizes after implantation in both sites.

## Discussion

Amount of studies have confirmed that the characteristics of scaffold including chemical composition, geometry shape, porosity, sizes and shapes of micro-/macro-pore can be tailored by varying processing parameters [41–43]. Previous researches have showed that macro-pore size of calcium phosphate scaffold was an important parameter to control cell response and bone formation [14]. Although some studies concluded that HA and tricalcium phosphate cylinders with the macro-pore sizes more than 100 μm was not requisite for bone ingrowth [28], others suggested that porous HA ceramics with macro-pore size of 300–400 μm was most effective for new bone formation and vascularization than other macro-pore sizes (e.g. 106–212, 212–300, 400–500 and 500–600 μm) [31, 32]. On the contrary, one study indicated that the osteoconduction of porous HA ceramics with macro-pore size of 175–260 μm was the best compared with macro-pore size of 260–350 and 350–435 μm [36]. However, other study indicated that the better cell colonization and a higher newly bone was found in biphasic calcium phosphate ceramics with macro-pore size of 565 μm than macro-pore size of 300 μm [33]. The β-tricalcium phosphate cylinders with different macro-pore sizes implanted into sheep suggested that a smaller of 260 μm or a larger pore size of 1220 μm might be more suited as bone alternate in animal model [44]. Therefore, there is little consensus about the optimal macro-pores sizes in porous scaffolds for bone formation, and many researches just focus on the change of macro-pores sizes and ignored the influence of interconnecting window diameter in macro-pores with different sizes in the process of bone formation. In this study, with the increase of macro-pores sizes, the interconnecting window diameter increased but the d/s ratio of window diameter to macro-pore size keep unchanged as about almost 0.26 (Table 1). All the HA scaffolds used for *in vivo* implantation had a similar interconnecting structure so that the 3D space seemed to be similar for cells and tissues to live in. In the other word, this study is focus on an absolutely similar porous structure only with three macro-pore sizes (Table 1 and Fig. 2) impacting tissue ingrowth and angiogenesis after *in vivo* implantation at non-osteo sites.

The diameter ratio of interconnecting pore to macro-pore in scaffold was very important for bone tissue formation. The macro-pore size was needed to match suitable interconnecting window diameter in scaffold for inducing new bone tissue formation. The space became lager with macro-pore size increasing, which required more invasion of blood vessel and tissue filled in. Thus the lager interconnecting windows was needed to provide the much wide pathway for protecting invasion of far more connective tissues and blood vessel. The size of interconnecting windows did not rise with macro-pores size increasing leads to the invasion of more connective tissues and blood vessel was limited, which against new bone fast formation. Bai *et al*. [35] found that macro-pore sizes and interconnecting window sizes both affected the angiogenesis of β-TCP scaffolds *in vivo*, and the interconnecting window affect not only the size of the blood vessels ingrowth but also the number of blood vessels formed in scaffold. Flautre et al revealed that four interconnecting windows size (30, 60, 100 and 130 μm) in porous HA ceramics with macro-pore sizes of 175–260 μm were implanted in rabbit femur cancellous bone defect after 12 weeks, the result found that macro-pore sizes of 175–260 μm with interconnecting windows size of 130 μm displayed the best osteconduction [36]. Feng *et al.* [45] suggested that β-tricalcium phosphate cylinders with four different macro-pore sizes and the same interconnecting windows size (120 μm) implanted into rabbits, which found that the smallest macro-pore sizes (300–400 μm) was beneficial for the growth of tissue and the diameter of pores lager than 400 μm have no evident difference on growth of blood vessels. Thus, to accurately compare the effect of macro-pores size of scaffold on blood vessel and bone tissue formation, the scaffold should possess similar interconnecting structure determined by the same ratio of interconnecting window diameter to macro-pore size.

The morphological observation of macro-porous structure in the three scaffolds (Figs 1 and 2) showed the uniform interconnected spherical pore shape similar to a trabecular bone [46]. The interconnecting window diameter was accurately tailored by adjusting the heat treat time on sugar sphere templates so that the ratio of window diameter to macro-pore size was kept unchanged for different macro-pore sizes, due to the contact of sugar spheres became from point-to-point to face-to-face under the heat treated process [47]. The porosities of all kinds of HA scaffolds (Table 1) met the clinical requirements of 50–90% for bone ingrowth [14]. There were no significant differences in the porosity for these scaffolds with different macro-pores; however, the compressive strengths of the three kinds of HA scaffolds decreased with increasing macro-pores sizes (Table 1 and Fig. 3A). The scaffolds with smaller macro-pores sizes had more struts per cube than those with larger macro-pores sizes that more struts could bear more compressive load [48, 49].

The abundant micro-pores existed on the wall of macro-pores benefit for protein absorption, cell adhesion and tissue growth by further increasing the surface roughness of the scaffolds [50, 51]. The micro-pores of the porous bioceramic played an important role in osteoinduction. The previous researches revealed that bone formation were observed in HA granules with micro-pores on their surface but not in HA granule without micro-pores under skin of dogs [52]. Yuan *et al*. [53] believed that apatite layer formation and protein adsorption on bioceramics surface closely related to bonding osteogenesis. The micro-pores on the surface of macro-pores wall enhanced highly the surface area which benefited for more proteins to be absorbed, and promoted exchanges of ion and formation of apatite layer by dissolution and reprecipitation process [54]. Furthermore, the rough surface created due to micro-pores existed on surface could accelerate osteogenic precursor cell adhesion, proliferation and differentiation [52, 53, 55, 56]. In addition, the micro-pores on macro-pores surface provide a suitable site of nucleation for this precipitation and induced bone tissue formation [51].

In addition, our previous researches demonstrated that macro-pore structure would influence fluid field distribution and fluid stress in the scaffold [38, 57]. The fluid dynamic environment in porous scaffolds will direct effect cell adhesion, migration, proliferation and differentiation, and hence influence the tissue ingrowth. The fluid swirl would be exists in scaffold with too low penetrability which prevented the exchange of metabolic substance and the interaction between cell and the surface of scaffold. Wang *et al.* [38] suggested that HA sphere-accumulated scaffolds with higher penetrability possessed much more stronger ability of inducing bone formation than porogen HA -negative scaffold with lower penetrability. In this study, the seepage flow of fluid went through the porous scaffold faster with macro-pores sizes increasing (Fig. 3C), due to increase of interconnecting window diameter. Thus, the fluid swirl would be exist due to the slower seepage flow in macro-pores sizes of 500–650 µm, which may be resulting in the interaction between cell and the surface of scaffold was reduced. On the contrary, the fluid in 1100–1250 µm probably was too quick for cell adhesion. The high shear force interfered with cell differentiation which also decrease new bone formation. Among the three HA scaffolds, the size of macro-pore in 700–950 µm leads to appropriate fluid velocity and shear force in the scaffold for osteogenesis (Fig. 3C), although the results of 3D cell culture showed that there were no significantly different about cell proliferation in scaffolds with three macro-pores sizes and even the pore structure of 500–650 µm seemed to facilitate cell growth in its macro-pores (Fig. 3B). This may be due to 3D cell culture was under static state which cannot simulate the dynamic flow environment in scaffolds after implantation.

Considering that ectopic implantation could be better proved the osteoinductivity of bioceramic scaffolds [38], the site of abdominal cavities and dorsal muscles was chosen to implant porous HA scaffolds with different macro-pores sizes. The histologist results showed that blood vessel and new bone formation in all scaffolds at different time points, and bone formation underlying a path akin to intramembraneous bone formation, as observed in skeletal formation (Figs 4 and 5). That is to say, osteogenic cells in scaffolds were in the presence of a good blood supply and adjacent to a local deposit of calcium salts to differentiate into bone cells [58–60]. Previous research revealed that the blood clot formed firstly and blood vessel invaded subsequently in macro-pores of ceramics after implantation [61]. In ectopic bone formation, vascularization was a key factor that provided oxygen, nutrient and signaling molecules for bone formation. Moreover, the blood vessel was a supply of progenitor cells which differentiated into osteoblasts due to the stimulation of the inflammatory cytokines [62]. Some studies indicated that pericytes from blood vessel could differentiate into osteoblasts [63]. And the osteoinduction has often been closely related to the existence of blood vessels in macro-pores of HA ceramics [62]. In addition, the release of calcium and phosphate ions from bioceramics cooperated with support of blood vessels and body fluids leads to the precipitation of biological apatite containing endogenous proteins on the surface of the ceramics [54]. The biological apatite layer attached by osteogenic cells, which produce an extracellular matrix including collagen, non-collagenous proteins, and growth factors such as BMP [62]. The growth factors induce the differentiation of mesenchymal stem cells into osteoblasts, and followed by the hematopoietic stem cells differentiated into osteoclasts [64]. Subsequently, the extracellular matrix produced by osteogenic cells mineralized by osteoblasts and formed a cement line. Finally, the apatite layer was reconstructed into bone-like tissue by osteoclasts.

Histomorphometric measurement (Fig. 9) showed that the blood vessel and new bone formation was observed in all scaffolds at different time points which implanted in abdominal cavities and dorsal muscles, but the scaffold with macro-pores sizes in range of 750–900 μm exhibited much more abundant new bone formation and higher the amount of vessels per square millimeter than other two kinds of macro-pores sizes. These results seemed to suggest that there exists an optimal range in macro-pore size of HA scaffolds which is in favor of bone formation and blood vessels ingrowth under the condition of the similar interconnecting structure. In addition, HE staining (Fig. 8) revealed that HA scaffolds with three macro-pore size ranges implanted in dorsal muscle after 3 months exhibited different new bone tissue distribution. The new bone uniformly formed throughout the whole scaffold with macro-pores sizes of 700–950 µm, which revealed that 700–950 µm is a suitable macro-pore size for blood vessels and tissue rapid filled the whole space and induced bone formation. In scaffold with macro-pores sizes of 500–650 µm, the new bone major distributed in peripheral region but not in the center region which may due to scaffold with too low penetrability prevented tissue successfully penetrated the center of scaffold. And the tissue occupied more pore space in peripheral region of scaffold result in reducing of the residual pore space for blood vessel ingrowth which against tissue formation subsequent. Although the scaffold with macro-pores sizes in1100–1250 µm exhibited new bone tissue growth in peripheral and center of scaffold, the speed of new bone formed was obviously slower than scaffold with macro-pores sizes in 700–950 µm, may due to the bigger macro-pores needed more blood vessels and tissue ingrowth to occupy all the space which limited the new bone fast formed in a short period of time [65].

## Conclusion

In this study, highly interconnected HA scaffolds with different macro-pores sizes and similar interconnecting structure were successfully fabricated by sugar spheres template leaching techniques. The scaffolds with different macro-pore sizes had approximately porosities of 83% while the compressive strength decreased with increasing macro-pore sizes. The *in vivo* experimental results clearly demonstrated that tissue ingrowth and blood vessels in scaffold closely related to their macro-pore sizes under the condition of similar interconnecting structure. Meanwhile, the neovascularization was critical factor during bone tissue formation in the scaffold. More angiogenesis results in more bone tissue formation. Among three HA scaffolds with the similar interconnecting structure, the amount of bone tissue and neovascularization in macro-pores sizes of 750–900 μm was more compared with smaller and larger macro-pores, which could assist in the optimization of porous scaffolds for bone regeneration.
